# Routine foreskin pathology in adults: Low yield, high cost—Time to change practice?

**DOI:** 10.1002/bco2.70299

**Published:** 2026-11-06

**Authors:** Kenneth A. McLean, Ellen Gardner, Kathryn Kirkwood, CJ Shukla, Alexander Laird, James P. Blackmur

**Affiliations:** ^1^ Department of Urology University of Edinburgh, Western General Hospital Edinburgh UK; ^2^ Centre for Medical Informatics, Usher Institute University of Edinburgh Edinburgh UK; ^3^ Department of Pathology Western General Hospital Edinburgh UK; ^4^ Institute of Genetics and Cancer University of Edinburgh Edinburgh UK

**Keywords:** circumcision, cost‐effectiveness, histopathology, number‐needed‐to‐screen, penile cancer

## Abstract

**Objective:**

This study aimed to determine the diagnostic yield for malignant lesions and cost‐effectiveness of routine foreskin pathology in adults.

**Subjects/Patients and Methods:**

A retrospective audit was conducted of all adult circumcisions (age ≥16) performed between January 2015 and February 2025 at a UK tertiary centre. Clinical and pathology records were reviewed to identify unexpected cancer diagnoses—defined as cases with no prior biopsy evidence or clinical suspicion. The number needed to screen (NNS) to detect one cancer was calculated, and pathology costs were estimated based on local processing costs and the pathologist workload score for each sample.

**Results:**

There were 2503 adult circumcisions performed between 2015 and 2025, with 2.6% (*n* = 66/2503) cancers detected on pathological assessment. Of these, 78.8% (*n* = 52/66) patients with confirmed penile cancer had clinically suspicious findings pre‐operatively. Therefore, the NNS on routine pathology for unexpected cancers was 109.4 (95% CI = 65.4–183.4), and only one clinically significant cancer (7.1%, *n* = 1/14 [0.04% overall cohort]) required further treatment (topical ablation). The diagnostic yield was also age‐dependent, with low diagnostic yields in patients below age 40 with potentially suspicious pathology (NNS = 178.5, 95% CI: 49.5–650.3). Pathology‐associated costs were estimated at £65 584 over the study period (£994 per cancer identified). Targeting pathology to men aged ≥40 with clinical suspicion of cancer would reduce costs by 73.3% (£48 074), while reducing the cost per cancer detected to £350.

**Conclusion:**

Routine foreskin pathology after adult circumcision has a low diagnostic yield for unexpected malignancy and rarely alters management. Targeting pathology to higher yield patient groups may optimise resource use, aligning with national guidelines and delivering substantial cost savings.

## 1 INTRODUCTION

Circumcision remains one of most common surgical procedures in the world, predominantly conducted in childhood for religious and cultural reasons.^1^ However, circumcision in adults remains an important treatment for a variety of benign conditions (including phimosis, balanitis, genital warts) and for suspected penile cancers. While there are no guidelines that mandate routine histopathological assessment of circumcision,^2^, ^3^ it has become widespread in clinical practice that adult foreskins are routinely sent for histopathology analysis. This reflects clinician concern regarding occult pre‐malignant or malignant lesions, which may not be apparent on clinical exam and which may necessitate additional treatment and/or follow‐up. However, current evidence would suggest the yield of penile cancer detection remains low in this circumstance, with the majority identified on clinical examination and/or preoperative biopsies.^4^, ^5^, ^6^, ^7^, ^8^, ^9^ Furthermore, circumcision is considered a definitive treatment for both benign conditions and malignancy confined to the foreskin.^2^, ^3^ Therefore, pathologic assessment is unlikely to change clinical management for these patients.

The questionable benefit of routine pathology in this context is becoming increasingly important to consider given the reported global shortage of pathology services, with clinical demand only expected to grow.^10^, ^11^ This already leads to delays in cancer diagnosis and treatment, risking poorer experience and outcomes for patients.^11^ Recent guidance from the Royal College of Pathologists (RCPath)^12^ has specifically highlighted circumcision specimens as an area of limited or no clinical value for routine pathology. To date, the few studies published on this topic have been small^4^, ^5^, ^6^, ^7^, ^8^, ^9^ and do not directly investigate the impact from rationalising routine pathology requests for adult circumcision. Therefore, this study aimed to evaluate the impact of a targeted approach to pathological assessment on the diagnostic yield and management of unexpected penile cancers and pathology‐related costs.

## 2 SUBJECTS/PATIENTS AND METHODS

### 2.1 Study design

This was a retrospective cohort study of all adult circumcisions (age ≥16) performed between January 2015 and February 2025 at a high‐volume tertiary centre in the United Kingdom. This excluded patients who were not based in the local region (NHS Lothian) at the point of referral, and circumcisions conducted as part of other procedures. This study was reported according to the ‘Strengthening the Reporting of Observational Studies in Epidemiology’ (STROBE) statement.^13^ Formal institutional ethical approval was not required as this study was considered a service evaluation, involving routinely collected data with no changes to clinical care pathways.

### 2.2 Data Sources

Electronic health records were reviewed across local operative and pathology databases to identify patients undergoing circumcision with foreskin specimens sent to pathology (ICD‐10 codes). Furthermore, data on the indication for circumcision (classified as phimosis, inflammation, growth or other^14^), and the rate and clinical management of penile cancers identified were extracted (including carcinoma‐in‐situ). The primary outcome of interest was the rate of clinically significant unexpected cancer. An unexpected cancer was defined as penile cancers detected on routine pathology with no prior imaging/biopsy evidence, or suspicion based on preoperative or intraoperative examination. ‘Clinical suspicion of cancer’ on exam encompassed any inflammatory change (red patch) or raised lesion (growth) noted on operation note, preceding clinic letter or pathology request. Clinically significant cancers were defined as those who went on to receive active treatment (whether local medical therapies or surgical procedures based on the multidisciplinary team [MDT] decision).

### 2.3 Statistical analysis

Numerical data were summarised as mean (standard deviation) or median (interquartile range) based on visual and statistical evaluation for normality, with appropriate tests for parametric or non‐parametric data performed. Categorical data were cross‐tabulated and tested using χ^2^ or Fisher's exact tests. Statistical significance was set a priori at *p* < 0.05, and all statistical analyses performed in R version 4.5.2 (R Foundation for Statistical Computing, Vienna, Austria). Any missing data were handled via exclusion from relevant analyses. A priori patient subgroups were explored based on age at time of procedure (decile) and indication for circumcision given their importance as risk factors for penile cancer.^15^

The numbers needed to screen (NNS) to detect one cancer was calculated as 1/proportion of cancers detected,^16^, ^17^ with binomial 95% confidence intervals (95% CI). Furthermore, pathology‐related costs were calculated on an individual basis and encompassed costs associated with processing and review of specimens. Each specimen had been assigned a pathologist workload score in line with RCPath guidelines, which is used to quantify the complexity and pathologist time required for review.^18^ This assigns points based on the time required with each point being equivalent to 5 min. Costs for pathologist time per sample were calculated by multiplying the full‐time equivalent proportion of pathologist time required per sample (assuming a 40 h work week year‐round) by the annual salary for a pathologist (assuming a first year consultant in NHS Scotland—£111 430 in 2024/25^19^). Furthermore, the estimated processing costs for foreskin pathology specimens were provided by the local pathology department.

## 3 RESULTS

### 3.1 Cohort characteristics

There was a total of 3593 circumcision specimens received by the local pathology unit between January 2015 and February 2025. There were 1090 patients excluded from the analysis due to being paediatric circumcisions (*n* = 466); referred from out of region (*n* = 623); and being incidentally performed as part of another procedure (*n* = 1).

Of the 2503 eligible procedures, the most common indications for circumcision were pathology that was not suspicious for cancer (61.2%, *n* = 1532/2503), including phimosis (61.0%, *n* = 1528/2503) and other benign indications (0.2%, *n* = 4/2503). In comparison, a substantial minority of circumcisions were deemed to have been performed for potential clinical suspicion of cancer (38.8%, *n* = 971/2503)—predominantly for red patches (35.2%, *n* = 881/2503) rather than growths (3.6%, *n* = 90/2503). The volume of circumcisions performed increased over time until the COVID‐19 pandemic and have yet to recover to pre‐pandemic levels (Figure 1A). Nevertheless, the relative distribution of indications for circumcision remained consistent (Figure 1B). The median age among the cohort was 50.0 although with significant variation (IQR = 29.0 to 65.0) (Table 1).

**FIGURE 1:**
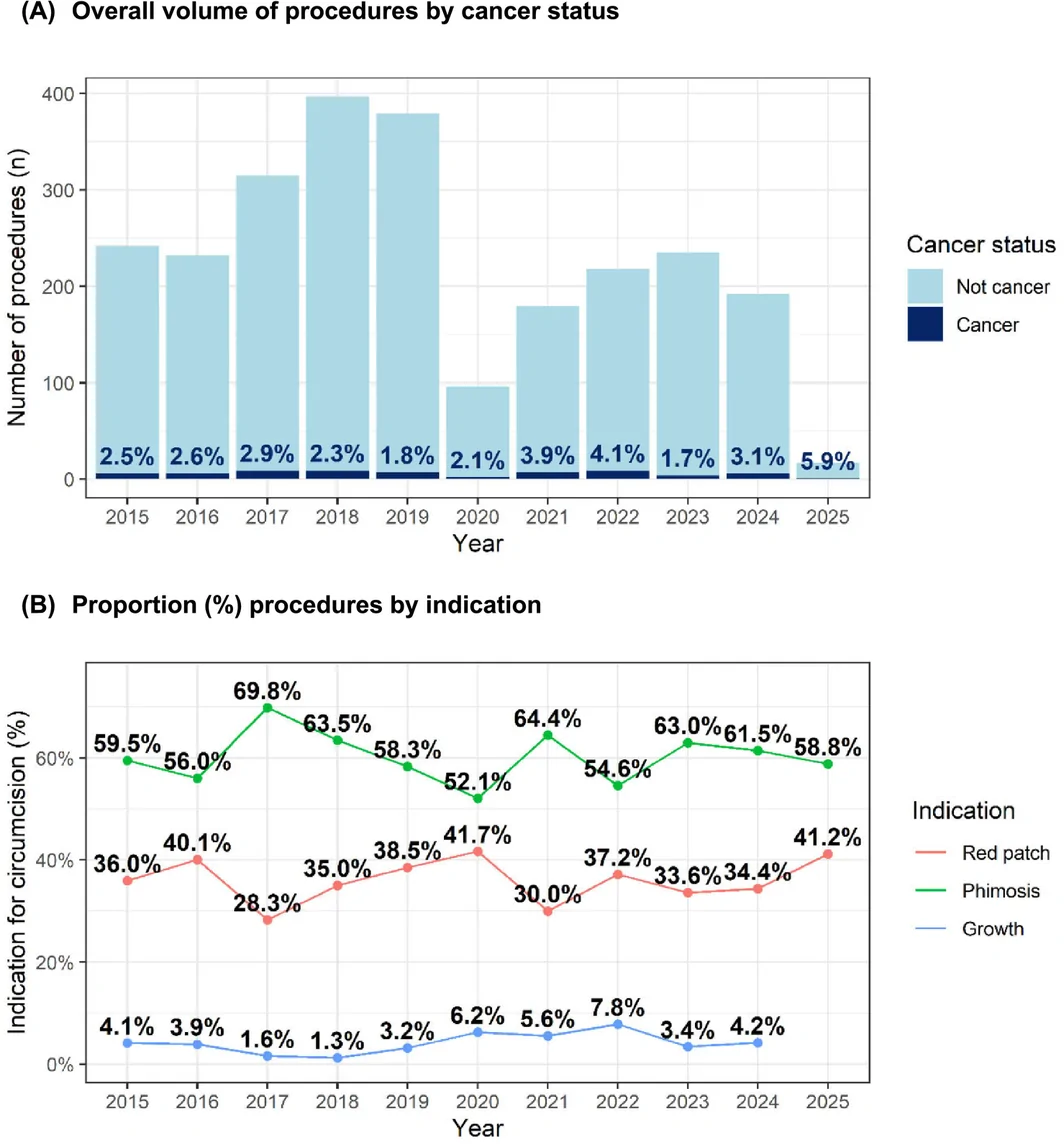
Temporal changes in operative volume and indication for circumcision in the cohort. (A) Overall volume of procedures by cancer status. (B) Proportion (%) procedures by indication.

**TABLE 1:** Cohort characteristics according to clinical suspicion of cancer.

		All patients (*n* = 2503)	No clinical suspicion of cancer (*n* = 1532)	Clinical suspicion of cancer (*n* = 971)
Age (years)	Median (IQR)	50.0 (29.0 to 65.0)	49.5 (29.0 to 65.0)	51.0 (28.0 to 66.0)
Indication	Phimosis	1528 (61.0)	1528 (99.7)	‐
	Red patch	881 (35.2)	‐	881 (90.7)
	Growth	90 (3.6)	‐	90 (9.3)
	Other	4 (0.2)	4 (0.3)	‐

### 3.2 Diagnostic yield for penile cancers

Across the cohort, there were 2.6% (*n* = 66/2503) penile cancers identified on pathological assessment. This remained consistent across the time‐period (Figure 1A). These were predominately identified in patients where the indication for circumcision was a growth (38.9% [*n* = 35/90], NNS = 2.6 [95% CI = 2.0–3.4]), with substantially lower diagnostic yields when performed for red patches (1.9% [*n* = 17/881], NNS = 51.8 [95% CI = 32.6–82.8]) or phimosis (0.9% [*n* = 14/1528], NNS = 109.1 [95% CI = 65.3–183.0]) (Table 2).

**TABLE 2:** Diagnostic yield (%) and number needed to screen (NNS) for penile cancer, by indication for circumcision.

		All cancer		Unexpected cancer		Clinically significant unexpected cancer	
		Diagnostic yield (%)	NNS (95% CI)	Diagnostic yield (%)	NNS (95% CI)	Diagnostic yield (%)	NNS (95% CI)
Overall		2.6% (*n* = 66/2503)	37.9 (95% CI = 29.9–48.1)	0.9% (*n* = 14/1532)	109.4 (95% CI = 65.4–183.4)	0.1% (*n* = 1/1532)	1532 (95% CI = 271.1–8678)
Indication	Phimosis	0.9% (*n* = 14/1528)	109.1 (95% CI = 65.3–183.0)	0.9% (*n* = 14/1528)	109.1 (95% CI = 65.3–183.0)	0.1% (*n* = 1/1528)	1528 (95% CI = 270.4–8655)
	Red patch	1.9% (*n* = 17/881)	51.8 (95% CI = 32.6–82.8)	‐	‐	‐	‐
	Growth	38.9% (*n* = 35/90)	2.6 (95% CI = 2.0–3.4)	‐	‐	‐	‐
	Other	0.0% (*n* = 0/4)	>4	0.0% (*n* = 0/4)	>4	0.0% (*n* = 0/4)	>4

The majority of cancers were known based on preceding biopsy results (33.3%, *n* = 22/66) and/or suspected based on clinical examination (45.5%, *n* = 30/66), meaning the diagnostic yield of unexpected cancers was 0.9% (*n* = 14/1532) from patients with a clinical impression of simple phimosis or other pathology (Table 2). In 14.3% (*n* = 2/14) cases of unexpected cancer, these phimoses coexisted with a histological diagnosis of Balanitis Xerotica Obliterans (BXO) (lichen sclerosus). Only 0.1% (*n* = 1/1532) of the cases of unexpected cancer were deemed clinically significant as they went on to have further treatment beyond the circumcision (topical ablative therapy to the glans). Therefore, while the NNS for unexpected cancers was 109.4 (95% CI = 65.4–183.4), this rose to 1532.0 (95% CI = 271.1–8678.0) to identify one clinically significant unexpected cancer.

The NNS to detect any penile cancers across the lifespan were also investigated. This remains high in young adults (under 40 years), however reduced substantially in older patients (Figure 2). This principally results from the higher diagnostic yield in patients with clinical suspicion of cancer, with the NNS significantly reducing from 178.5 (95% CI: 49.5–650.3) in those under age 40 to 12.3 (95% CI: 9.5–16.0) in those over age 40.

**FIGURE 2:**
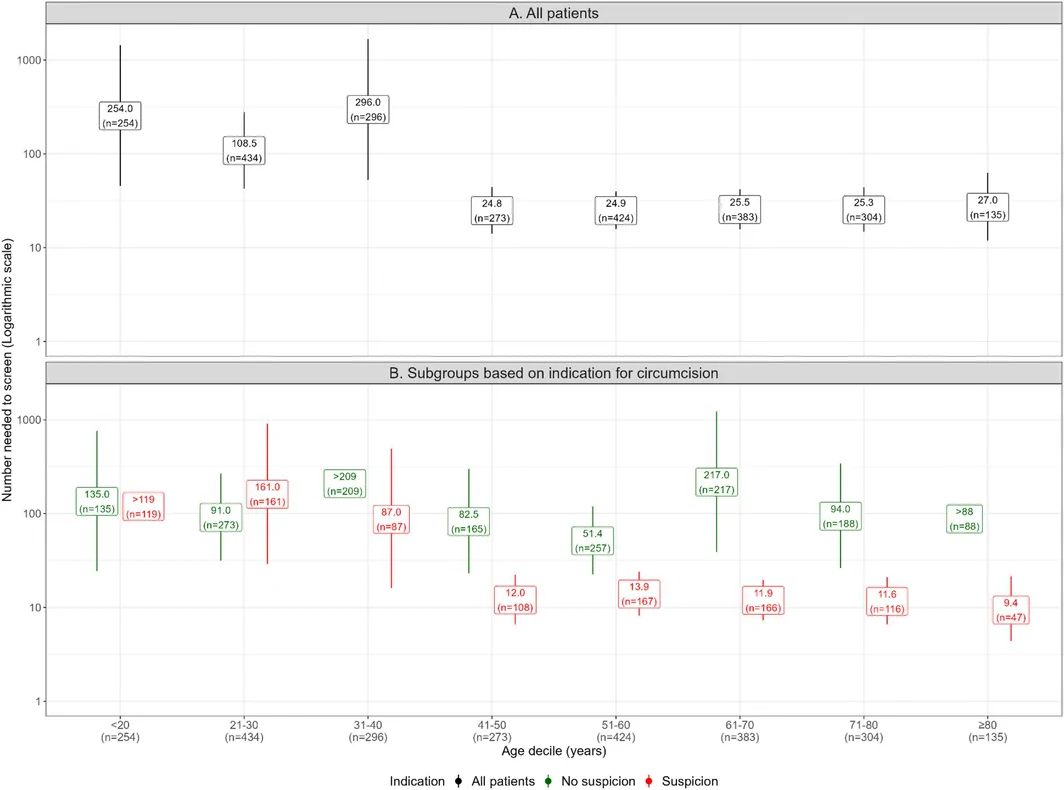
Number needed to screen (NNS) for any penile cancers, by age decile and indication for circumcision.

### 3.3 Pathology‐associated costs

The overall pathology‐associated cost over the study period was estimated to be £65 584 (mean annual cost = £6491), approximating £994 per cancer identified (Table 3). Of this, 59.2% (£38 833) of pathology‐associated costs related to specimens where there was no clinical suspicion (£2774 per cancer identified) and 14.1% (£9241) to men under 40 with clinical suspicion (£4620 per cancer identified). Within this cohort, targeting pathology towards older men with suspicion of cancer (red patch or growth) was estimated to reduce pathology‐associated costs by 73.3% (£48 074) reducing the cost per cancer identified to £350.

**TABLE 3:** Economic impact of rationalising routine pathology for circumcision to identify any penile cancer, by clinical subgroup.

	Pathology‐associated cost (£)	Diagnostic yield (%)	NNS	Cost per cancer (£)
Overall cohort	£65 584	2.6% (*n* = 66/2503)	37.9	£994
No clinical suspicion	£38 833 (59.2%)	0.9% (*n* = 14/1532)	109.4	£2774
Clinical suspicion	£26 751 (40.8%)	5.4% (*n* = 52/971)	18.7	£514
Age < 40	£9241 (14.1%)	0.6% (*n* = 2/357)	178.5	£4620
Age ≥ 40	£17 510 (26.7%)	8.1% (*n* = 50/614)	12.3	£350

## 4 DISCUSSION

There is increasing evidence that routine pathological assessment across many surgical procedures is often not cost‐effective,^20^ with guidelines starting to reflect this.^12^ In this large retrospective cohort study of adult circumcision specimens submitted for histopathological assessment over a 10‐year period, the overall detection rate of penile cancer was 2.6% (*n* = 66/2503). However, the diagnostic yield was indication‐ and age‐dependent, with low diagnostic yields observed in patients with phimosis in the absence of other worrying feature (0.9%; NNS = 109.1) or below age 40 even with potentially suspicious pathology (0.6%, NNS = 178.5). Critically, after accounting for cases already biopsy‐proven or clinically suspected (documented red‐patch or growth), the rate of unexpected malignancy was low (0.9%; NNS = 109.4). Only a single case (0.1%, NNS = 1532) required further treatment and was considered a clinically significant unexpected cancer. Modelling a targeted strategy—restricting histology to older men with clinical suspicion—estimated a 75% reduction in pathology‐associated costs (~£48 000) and a marked reduction in cost per cancer detected (over £3000 in non‐prioritised patients to £350 in prioritised patients). If these results are applied to England in 2024–25^21^ (21 172 adult circumcisions), over £400 000 in pathology‐associated costs could be potentially avoided annually.

The published evidence with regard to adult circumcision remains limited and geographically restricted, predominantly being conducted in high‐income settings (Table S1). Across these studies, the proportion of specimens routinely processed varies widely (median = 85.3%, range = 20.2%–100%). This introduces a risk of selection bias, and as such, the studies with incomplete pathological assessment may overestimate the diagnostic yield and therefore the benefit of routine pathological assessment of the prepuce post‐circumcision.^4^, ^7^, ^8^ Nevertheless, the diagnostic yield of any penile cancer in this study (2.6%, *n* = 66/2503) was consistent with previous publications (median = 2.8%, range = 0.9%–5.9%). Moreover, across all previous studies, there were no clinically significant unexpected cancers reported (0%). This aligns with these results (0.1%, *n* = 1/1532) and supports the view that routine pathology rarely adds actionable information. Only two previous studies have explored pathology‐related costs, reporting inflation‐ and currency‐adjusted average costs per specimen of £64^8^ (£34.33 in England in 2008) and £320^4^ ($311 in the United States in 2013). While in this context, the cost per specimen was lower than previous estimates (£26), it should be noted the cost per cancer identified was substantial (£994). Moreover, the cost to identify the single clinically significant unexpected cancer was £65 600 **(**Table 3 **)**. Furthermore, none have explored the impact on rationalisation of pathologic assessment on these costs, with this study being able to demonstrate significant cost reductions for targeted approaches, including omitting for patients with simple phimosis (59.2%) and aged less than 40 with potentially suspicious pathology (14.1%). Nevertheless, it should be noted that there remain important subgroups at higher risk for penile cancer (e.g., patients known to have HIV or HPV infections)^15^ or high suspicion on clinical examination, and as such warrant a lower threshold of suspicion to request pathological assessment.

Prior to this study, the published evidence has consistently remained against routine pathology for adult circumcision, yet this practice remains widespread. There has been no specific research into the underlying surgical decision making for requesting pathological assessment in the case of adult circumcision. However, the underlying drivers of clinicians ordering ‘low‐value’ diagnostic tests have been studied across a range of contexts.^22^, ^23^, ^24^, ^25^ Often clinicians will recognise potential low yield tests; however, the fear of missing a clinically significant diagnosis, even in the absence of clinical suspicion, remains a strong motivator. In many surgical procedures, benign pathology can nonetheless have clinical relevance even without a risk of cancer. However, in the case of circumcision, these are frequently curative of the underlying pathology with no requirement for further management.^2^, ^3^ Nevertheless, organisational, sociocultural and patient expectations often prioritise avoidance of harm rather than necessarily the pursuit of benefit.^22^, ^23^, ^24^, ^25^ Furthermore, this can also be driven by the desire to reassure patients, even when the diagnostic yield is low.^26^ This may reflect that pre‐test probability is a concept that is frequently misunderstood and overestimated by clinicians,^27^, ^28^ and so justifying to patients why pathological assessment may not be required may be considered challenging. It should also be noted this practice may also not be successful at reassuring patients. Overdiagnosis of clinically insignificant cancers is not without its consequences, with the negative psychosocial impact well documented in the context of prostate cancers.^29^ Finally, the workload and costs entailed with pathological assessment are often not directly borne by the requesting surgeon, meaning the marginal gain may still be considered of benefit. Ultimately, the de‐implementation of low‐yield pathological assessment will not succeed without addressing these underlying drivers. Evidence from other contexts suggests that clear evidence‐based clinical guidelines on the indications for pathological assessment, together with patient educational materials are needed to drive such change.^26^

There are several notable strengths to this study. Firstly, this study represents the largest dataset on this topic to date (Table S1). This is important given the relatively low prevalence of unexpected penile cancer observed previously, raising confidence that clinically significant cancers are unlikely to be missed in rationalisation. Furthermore, this study provides detailed analysis of the impact of rationalisation according to published pathology guidelines,^12^ directly addressing clinical concerns regarding the potential for missed clinically significant cancers (Table 2). By providing the NNS across age groups, this allows a clearer understanding regarding the risk of malignancy to inform clinical decision making. However, there were also several important limitations. Firstly, while this study was conducted at a high‐volume tertiary centre, this remains a single centre study and so care should be taken when generalising to different populations and health systems. In particularly, countries with high rates of cultural or religious circumcision in childhood.^1^ However, the local clinical practice is reflective of known practice around routine pathology for circumcision specimens with penile cancer detection rates in line with published evidence (Table S1). Therefore, while the precise pathology‐related costs may vary according to the location, the underlying potential for a reduction in pathology‐related costs and workload would be expected to remain consistent. Secondly, the cost‐effectiveness results only focus on the direct pathology‐related costs. This does not consider downstream costs from missed clinically‐significant cancers which would affect patients and health systems, but equally does not consider costs related to unnecessary follow‐up and MDT meetings which may increase cost‐effectiveness. It also does not encompass indirect benefits such as increased turnaround of other higher yield pathological specimens, enhancing cancer care pathways. Finally, a conservative definition of ‘clinical suspicion of cancer’ was used to encompass any inflammatory change (red patch) or growth under the assumption that the pathological assessment of specimens was performed for investigation of cancer. There is evidence in this (Table 2) and other studies (Table S1), which support that many penile cancers are accurately identified on clinical exam. Therefore, some of these cancers may have been considered unexpected had the clinical opinion based on exam been formally incorporated. This was not routinely recorded at this centre, and so was not possible to directly investigate within this retrospective study. Nevertheless, this definition is reflective of current practice given the routine nature of pathology requests.

Overall, this study demonstrates that routine pathological assessment of foreskin specimens after adult circumcision has a low diagnostic yield for unexpected malignancy and rarely alters management. Targeting pathology to higher yield patient groups would be expected to optimise resource use, align with national guidelines and deliver substantial cost savings. However, it should be recognised that these potential benefits depend on behavioural change of long‐established practice. Therefore, an international consensus process to develop clear guidance on when circumcision specimens can be safely omitted from pathological assessment would support evidence‐based practice, reassure clinicians and patients, and help ensure limited pathological capacity is prioritised for clinically significant pathology.

## AUTHOR CONTRIBUTIONS

**Kenneth A. McLean** and **Ellen Gardner:** Conceptualization; methodology; data curation; formal analysis; investigation; visualisation; writing. **Kathryn Kirkwood** and **CJ Shukla:** Data curation and manuscript review. **Alexander Laird** and **James P. Blackmur:** Conceptualization; supervision; methodology; critical revision of the manuscript.

## CONFLICT OF INTEREST STATEMENT

None to declare.

## Supporting information


**Table S1:** Publications to date on routine pathology for penile cancer in adult circumcision.

## Data Availability

The data that support the findings of this study are available from NHS Lothian. Restrictions apply to the availability of these data, which were used under license for this study. Data are available from the author(s) with the permission of NHS Lothian.
