# Supplementary material for: Routine foreskin pathology in adults: Low yield, high cost—Time to change practice?
Source: BJUI Compass. 2026 Nov 6;7(11):e70299. doi: 10.1002/bco2.70299 (PMC13743916; doi:10.1002/bco2.70299)
Supplement: Supplementary file 1 [file BCO2-7-e70299-s001.docx]

**Supplement**

**Supplementary Table 1: Publications to date on routine pathology for penile cancer in adult circumcision**

|  |  | **Proportion of specimens sent for pathology (%)** | **Penile cancers identified on pathological assessment** | | |
| --- | --- | --- | --- | --- | --- |
| **Publication** | **Location** |  | **All cancers (%)** | **Unexpected cancers (%)** | **Clinically-significant unexpected cancers (%)** |
| Pearce (2002) ^8^ | UK | 20.2 (n=93/460) | 4.3 (n=4/93) | 2.2 (n=2/91) | 0 (n=0/91) |
| McSorley (2011) ^9^ | UK | 100 (n=51/51) | 5.9 (n=3/51) | 0 (n=0/48) | 0 (n=0/48) |
| Shah (2015) ^4^ | US | 68.7 (n=101/147) | 1.0 (n=1/101) | 1.0 (n=1/101*) | 0 (n=0/101) |
| Safir (2015) ^5^ | US | 100 (n=225/225) | 4.0 (n=9/225) | 0 (n=0/209) | 0 (n=0/209) |
| Kerr (2020) ^7^ | UK | 70.6 (n=359/508) | 1.7 (n=6/359) | 0 (n=0/353) | 0 (n=0/353) |
| Khalabazyane (2024) ^6^ | UK | 100 (n=334/334) | 0.9 (n=3/334) | 0 (n=0/325) | 0 (n=0/325) |

* Occurred in the context of an HIV positive patient with simple phimosis
